# A Guide to the Qualitative and Quantitative Analysis of the Urine

**Published:** 1879-07

**Authors:** 


*A Guide to the Qualitative and Qantitative Analysis of the Urine, designed for Physicians, Chemists and Pharmacists.* By Dr. C. NEUBAUER and J. VOGEL, with a preface by Prof. Dr. R. FRESENINS.

The great importance of Urinary Chemistry is being more and more fully impressed upon the medical student and practitioner, and we believe there is no book in the English language which treats the subject in so thorough and scientific a manner as the one before us. It is separated into two distinct parts, the first being Chemical and the second chiefly Medical, adds to its value as a book of reference for both chemists and physicians. The whole subject is presented in this work and brought up to the present state of knowledge in this important and progressive branch of study.

The style of publication attracts our especial admiration, instead of the light colored leather of former fashion so easily soiled, we have a red stained calf, which seems to us a great improvement on former styles, and we hope it will be generally adopted if proved to be more desirable, as certainly it would at present appear to to be. Time however, is the test of seeming improvements. Meanwhile we congratulate Mr. Wood & Company upon the prospect.

